# Factors Associated with Willingness to Accept Oral Fluid HIV Rapid Testing among Most-at-Risk Populations in China

**DOI:** 10.1371/journal.pone.0080594

**Published:** 2013-11-19

**Authors:** Huanmiao Xun, Dianmin Kang, Tao Huang, Yuesheng Qian, Xiufang Li, Erin C. Wilson, Shan Yang, Zhenxia Jiang, Cuihua Gong, Xiaorun Tao, Xijiang Zhang, Guoyong Wang, Yapei Song, Zhijian Xu, Gifty Marley, Pengcheng Huai, Wei Ma

**Affiliations:** 1 School of Public Health, Shandong University, Jinan, China; 2 Shandong Provincial Center for Disease Control and Prevention, Jinan, China; 3 The Affiliated Hospital of Medical College Qingdao University, Qingdao, China; 4 San Francisco Department of Public Health, San Francisco, California, United States of America; 5 Yantai Municipal Center for Disease Control and Prevention, Yantai, China; 6 Qingdao Municipal Center for Disease Control and Prevention, Qingdao, China; 7 Zibo Municipal Center for Disease Control and Prevention, Zibo, China; UNC Project-China, China

## Abstract

**Background:**

The availability of oral fluid HIV rapid testing provides an approach that may have the potential to expand HIV testing in China, especially among most-a-risk populations. There are few investigations about the acceptability of oral fluid HIV testing among most-at-risk populations in China.

**Method:**

A cross-sectional study with men who have sex with men (MSM), female sex workers (FSW) and voluntary counseling and testing (VCT) clients was conducted in three cities of Shandong province, China from 2011 to 2012. Data were collected by face-to-face questionnaire.

**Results:**

About 71% of participants were willing to accept the oral fluid HIV rapid testing, and home HIV testing was independently associated with acceptability of the new testing method among MSM, FSW and VCT clients (AOR of 4.46, 3.19 and 5.74, respectively). Independent predictors of oral fluid HIV rapid testing acceptability among MSM were having ever taken an oral fluid HIV rapid test (AOR= 15.25), having ever taken an HIV test (AOR= 2.07), and education level (AOR= 1.74). Engagement in HIV-related risk behaviors (AOR= 1.68) was an independent predictor of acceptability for FSW. Having taken an HIV test (AOR= 2.85) was an independent predictor of acceptability for VCT clients. The primary concern about the oral fluid HIV testing was accuracy. The median price they would pay for the testing ranged from 4.8 to 8.1 U.S. dollars.

**Conclusion:**

High acceptability of oral fluid HIV rapid testing was shown among most-at-risk populations. Findings provide support for oral rapid HIV testing as another HIV prevention tool, and provide a backdrop for the implementation of HIV home testing in the near future. Appropriate pricing and increased public education through awareness campaigns that address concerns about the accuracy and safety of the oral fluid HIV rapid testing may help increase acceptability and use among most-at-risk populations in China.

## Introduction

In China, the most-at-risk populations for HIV infection have been changing over time, from injection drug users (IDUs) and plasma donors to heterosexuals contracting HIV via commercial sex with female sex workers (FSW) and homosexual transmission among men who have sex with men (MSM) [1]. National HIV/AIDS reports in China found that sexual transmission accounted for 63.9% of the estimated 780,000 people living with HIV/AIDS (PLHIV) in 2011, up from 59% in 2009. Among them, 52.2% and 29.4% of all new HIV infections in 2011 were through heterosexual and homosexual transmissions, up from 44.3% and 14.7% in 2009 respectively [2]. FSW and MSM are not only passively impacted by HIV infection, but also important drivers of the HIV epidemic and should be regarded as most-at-risk and target population for HIV/AIDS prevention and control. However, prevention approaches are heavily dependent on knowledge of one’s HIV status. 

Voluntary counseling and testing (VCT) provides an opportunity for both primary prevention (i.e. preventing HIV-negative people from contracting the infection) and secondary prevention (i.e. avoiding the progression of the disease among infected people by providing early health care and psychosocial support), as it encompasses counseling before and after HIV testing [3]. A cumulative 343,000 PLHIV were identified at the end of September 2011, while it is estimated that there are 780,000 people in China living with HIV/AIDS [2]. Based on the estimates, almost half of the people living with HIV/AIDS have not yet been tested and diagnosed, thus there remains a high risk for onward transmission. Evidences till date supports the notion that early detection and diagnosis expedites early treatment and linkage to care, and also promotes reduction in HIV-related risk behaviors [4,5,6,7,8].Thus, early diagnosis may additionally aid in prevention of HIV transmission and provide an important public-health benefit [9].

Free VCT began being offered in China in 2003 as part of a comprehensive treatment program [10]. In order to improve HIV testing rate and encourage awareness of HIV status, the Chinese government has established free VCT clinics [1,11,12]. By 2008, more than 6,000 VCT clinics had opened nationwide [13], which increased from 4,293 VCT clinics in 2007 [4]. In high epidemic areas of China, the HIV positive rate was 66.7% among VCT clients with 94.7% having drug injection history and 83.3% being men who have sex with men [14]. According to a recent study among VCT clients in 2011, 553 out of 561 clients had high HIV-related risk behaviors, like having sex with MSM, donating plasma and occupational exposure (being pierced by needle or sharp devices polluted by blood or other body fluids containing HIV) [15]. Hence, VCT clients in China are categorized as most-at-risk population. However, low prevalence of HIV testing presents an important barrier to the scale-up of HIV prevention and treatment. 

Studies have identified a number of reasons why individuals do not test for HIV including: perceived discomfort, pain and fear of blood that comes with traditional blood-based tests [5,16,17,18]; concerns about confidentiality and high visibility; emotional vulnerability of receiving results; unwillingness to learn testing results in public; high standard of counseling required for HIV testing; stigma and discrimination [4,10,19,20,21]. 

In 2004, the US food and drug administration (FDA) approved the first rapid oral fluid testing for HIV to avoid some of these common barriers to HIV testing. Oral fluid rapid HIV testing was implemented because it was a simple, user friendly, accurate and convenient point-of-care HIV testing, with the possibility of obtaining test results within 20 minutes [22]. Typical barriers to HIV testing are avoided because the oral fluid HIV rapid testing offers individuals a chance to know their status in the privacy of their homes, ensuring confidentiality,promoting proactivity in healthcare decisions, avoiding the issue of stigma and visibility in public settings, and providing an opportunity for early diagnosis [23]. In addition, oral fluid rapid HIV testing offer several advantages over blood samples, such as ease of sample collection, better compliance for sample acquisition, reduction of occupational risk associated with needle stick injuries, low load of infectious virus in oral fluids, and safer disposal of waste materials [24]. Rapid point-of-care HIV testing is a very important component of HIV control initiatives and programs. In particular, non-invasive, simple, accurate oral fluid-based rapid HIV testing has the potential to make a big impact on HIV screening programs, especially in areas where laboratory infrastructure is poor or unavailable. And it also opens the possibility of home-based HIV testing [25].

In addition to the ease of use, some studies have proved that oral fluid HIV rapid testing is highly accurate and acceptable. For example, a study conducted in a rural hospital in Sevagram, Maharashtra found the oral fluid-based rapid HIV1/2 test was highly accurate [5]. When compared to a reference standard of ELISA and Western Blot, the estimated sensitivity and specificity was 100 per cent among 450 patients with suspected HIV [5]. In comparison to blood-based rapid testing and conventional testing (i.e. ELISA and Western Blot), the oral fluid-based testing was the most preferred testing by the study participants [5]. Despite the fact that diagnostic accuracy and positive-predictive values vary with prevalence of diseases [26], and concerns about affordability and the potential for misuse of results, there is still strong support for oral fluid HIV rapid testing. Although there was recent rash of false positive results, officials emphasized that the test remains an excellent screening tool, and many experts argue that the only way to halt the spread of the AIDS epidemic is to make HIV tests as simple as home pregnancy tests [25]. 

So far, the China FDA has approved 3 oral fluid HIV rapid testing kits using DOT-ELISA or Colloidal gold method. The first one was approved in March, 2008. The other two testing kits were approved in February and April, 2011. However, oral fluid HIV rapid testing has not yet been widely used in China, and to our knowledge, there are few investigations about acceptance of oral fluid HIV rapid testing among most-at-risk populations (including MSM, FSW and VCT clients). This study seeks to assess the willingness to accept the oral fluid HIV rapid testing and its associated factors among most-at-risk populations in Shandong province of China. 

## Methods

### Study design and participants

From July 2011 to December 2012, we conducted an investigation about acceptance of oral fluid HIV rapid testing among 3 most-at-risk populations in 4 cities of Shandong province in China. Men who have sex with men (MSM), female sex workers (FSW) and VCT clients were recruited from Qingdao City, Yantai City, Zibo City and Jiaozhou County of Qingdao. Staffs in local Center of Disease Control and Prevention (CDC) and VCT clinics, and doctors from local hospitals conducted the questionnaires. For MSM and FSW, most participants were recruited from bathhouses, massage centers, salons (FSWs), bars (MSM), hotels, and restaurants. Some MSM were recruited in workshops convened for AIDS education or the office of a local nongovernmental organization run by MSM. Potential participants were approached either through managers of their workplace or through organizers of workshops. VCT clients were recruited from VCT clinics in Qingdao City and Yantai City.

Inclusion criteria for the study were as follows: all participants had to be 18 and above years of age, willing to participate in the survey, had sex (either anal or oral or both) with other men in the past 12 months or provided commercial sex in the past 12 months (for FSW), or visited VCT clinics for HIV counseling and/or testing (for VCT clients). Willing participants who were intoxicated or mentally ill to the extent that they could not consent to study participation were excluded. 

### Data Collection

A pre-tested questionnaire was administered face-to-face to all participants. The information collected by the structured questionnaire included perceptions of and willingness to accept oral fluid HIV rapid testing. Questions about knowledge and willingness included: (1) have you ever heard of oral fluid HIV rapid testing?, (2) have you ever taken an HIV test in the past year?, (3) are you willing to accept oral fluid HIV rapid testing?, and (4) what are the reasons you are willing or unwilling to use the oral fluid HIV rapid test? Subjects were also asked if they were willing to personally pay for the oral fluid HIV rapid testing, if they thought VCT clinics should use oral fluid HIV rapid test, and whether or not subjects would prefer to take HIV testing at home using oral fluid test kits.

The questionnaire assessed basic social-demographic characteristics such as age, gender, education level, occupation, and monthly income. And it also assessed HIV-testing histories including whether subjects had ever taken HIV testing, what HIV testing methods subjects experienced, and the test results of the last HIV test. HIV-related risk behaviors in the last 3 months were assessed including drug injection history , donating plasma, blood transfusion, occupational exposure, surgery, anal sex without condom, heterosexual behavior without condom, and having had a HIV-positive spouse or sexual partners. Occupational exposure in this context refers to that laboratory and healthcare personnel who may have been exposed to blood and other body fluids of people living with HIV, patients with advanced acquired immune deficiency syndrome or have been pierced by a needle and sharp devices contaminated by blood or other body fluids containing HIV. Informed consent was obtained from each participant before the administration of the questionnaire. 

### Data Analysis

All the data were double entered with EpiData3.1 (The EpiData Association Odense, Denmark) and discrepancies were checked against the raw data. Data analysis was performed with Statistical Analysis System (SAS 9.1 for Windows; SAS Institute Inc., NC, USA). Chi-square tests, one-way analysis of variance (ANOVA) and logistic regression were used for data analysis. Chi-square tests were used to explore whether there were significant differences in demographic characteristics, perceptions and needs for oral fluid HIV rapid testing among MSM, FSW and VCT clients. One-way analysis of variance was used to analyze whether there were significant differences between the three groups. Univariate logistic regression analysis was performed to evaluate associations of willingness to use oral fluid HIV rapid test with socio-demographic characteristics, sexual behaviors and HIV testing in the three groups separately. Odds ratios (OR) and 95% confidence intervals were calculated accordingly. Variables with a *P* value < 0.10 in the univariate analysis were eligible for entry into the multivariable logistic regression model, where adjusted OR and 95%CI were calculated accordingly. A stepwise approach was used to build the final multivariable logistic regression model for each group. Statistical significance was defined as *P*<0.05 (two-tailed test).

### Ethical Statement

This study protocol was approved by the Institutional Review Board of Shandong University School of Public Health. Study procedure, voluntary nature of participation, participants' right to withdraw and autonomy of the participants were explained and written informed consent was obtained from the participants before their interviews.

## Results

### Socio-Demographics

A total of 1151 participants were recruited for the study. However, 4 MSM, 3 FSW and 7 VCT clients refused participation or could not complete the questionnaire. A total of 1137 participants were included in the analysis. There were 371 men who have sex with men (MSM), 405 female sex workers (FSW) and 361 voluntary counseling and testing (VCT) clients with participation rates of 98.9%, 99.3% and 98.1% respectively. Nearly four fifths of participants were male among VCT clients. The median age was 26 years (interquartile range: 23-31 years), 25 years (interquartile range: 23-28 years) and 28 years (interquartile range: 25-33 years) among MSM, FSW and VCT clients respectively. Over 60% of VCT clients had attended college, followed by MSM (56.3%) and FSW (4.9%). Only ten percent of MSM or VCT clients had a monthly income more than $645 USD, whereas nearly half of FSW earned more than $645USD per month. Business service workers, students, workers and food beverage workers were the primary occupations among MSM and VCT clients; 87.2% FSW worked on business service (Table 1).

**Table 1:** Comparison of demographic characteristics among MSM, FSW and VCT clients in Shandong, China (RMB: USD) = 6.2:1.

Characteristics	Most-at-risk population			χ^2^	P-value
	MSM	FSW	VCT clients		
Gender					
Male	371(100.0)	0(0.0)	284(78.7)	888.95	<0.001
Female	0(0.0)	405(100.0)	77(21.3)		
Age(years)					
≤25	198(53.4)	264(65.2)	130(36.0)	65.47	<0.001
>25	173(46.6)	141(34.8)	231(64.0)		
Educational level					
High school or lower	162(43.7)	385(95.1)	138(38.2)	320.64	<0.001
College or higher	209(56.3)	20(4.9)	223(61.8)		
Monthly income(USD)					
≤645	334(90.0)	204(50.4)	322(89.2)	218.02	<0.001
>645	37(10.0)	201(49.6)	39(10.8)		
Occupation					
Business service	81(21.8)	353(87.2)	64(17.7)	519.68	<0.001
Student	77(20.8)	3(0.7)	64(17.7)		
Workers	75(20.2)	3(0.7)	54(15.0)		
Food and beverage workers	61(16.4)	33(8.1)	95(26.3)		
Cadres staff	19(5.1)	0(0.0)	29(8.0)		
Teacher	8(2.2)	4(1.0)	10(2.8)		
Nanny/housewife/unemployment	8(2.2)	4(1.0)	10(2.8)		
Farmer/fisher/migrant workers	15(4.0)	3(0.7)	9(2.5)		
Others	27(7.3)	6(1.5)	24(6.6)		
HIV risk behavior					
Yes	330(88.9)	266(65.7)	313(86.7)	80.46	<0.001
No	41(11.1)	139(34.3)	48(13.3)		

### Willingness to accept oral fluid HIV rapid testing

Most participants were unfamiliar with the oral fluid HIV rapid testing: 45.6% of MSM had ever heard of it followed by VCT clients with 33.5% and FSW with no more than one tenth. A majority of MSM and FSW had ever taken an HIV test, but only 79 participants had ever taken the oral fluid HIV rapid test. About 71% (806/1137) of participants were willing to accept the oral fluid HIV rapid testing and the acceptance rate among MSM, FSW and VCT clients was 72.8%, 72.1% and 67.4% respectively. Nearly half of MSM had ever considered HIV home testing followed by one third of VCT clients and one fifth of FSW. Over half (54.3%) of most-at-risk populations considered taking home tests using oral fluid HIV testing kits (Table 2).

**Table 2:** Perceptions for oral fluid HIV rapid testing among MSM, FSW and VCT clients in Shandong, China.

Characteristics	Most-at-risk population			χ^2^	P-value
	MSM	FSW	VCT clients		
Having ever heard of oral fluid HIV rapid testing					
Yes	169(45.6)	39(9.6)	121(33.5)	126.91	<0.001
No	202(54.4)	36(90.4)	240(66.5)		
Having ever taken an oral fluid HIV rapid test					
Yes	48(12.9)	13(3.2)	18(5.0)	31.49	<0.001
No	323(87.1)	392(96.8)	343(96.8)		
Willing to accept oral fluid HIV rapid testing					
Yes	270(72.8)	292(72.1)	244(67.4)	2.83	0.243
No	101(27.2)	113(27.9)	117(32.4)		
Having ever taken an HIV testing					
Yes	249(67.1)	238(58.8)	153(42.4)	47.06	<0.001
No	122(32.9)	167(41.2)	208(57.6)		
Having ever considered HIV home testing					
Yes	177(47.7)	80(19.8)	116(32.1)	68.76	<0.001
No	194(52.3)	325(80.2)	245(67.9)		
Considering HIV home testing using oral fluid HIV test kits					
Yes	216(58.2)	207(51.1)	194(53.7)	4.00	0.135
No	155(41.8)	198(48.9)	361(46.3)		

For MSM, the most frequent reasons cited for willingness to accept oral fluid HIV rapid testing were that the test did not require a blood draw, the test was government approved, and there was no pain associated with getting tested. No blood draw and painlessness were the main reasons FSWs found the test acceptable. Not being a blood draw and quick test results were the reasons most VCT clients found the test acceptable. Both never having heard of the oral fluid HIV rapid testing and testing accuracy were concerns for MSM, FSW and VCT clients. Center for Disease Control and Prevention (CDC) was the most preferred test sites for MSM and VCT clients and hospitals specialized in HIV/AIDS were the best test sites for FSW. The median price that MSM, FSW and VCT clients were willing to pay for the oral fluid HIV rapid testing was $6.5 USD, $4.8 USD and $8.1USD respectively (Table 3).

**Table 3:** Needs for oral fluid HIV rapid testing among MSM, FSW and VCT clients in Shandong, China.

Characteristics	Most-at-risk population			χ^2^	P-value
	MSM	FSW	VCT clients		
Reasons for willing to accept oral fluid HIV rapid testing					
Approved by government	84(40.4)	40(22.5)	52(29.4)	14.745	<0.001
Common use	17(8.20	28(15.7)	13(7.3)	8.37	0.015
Accurate and reliable	63(30.3)	26(14.6)	25(14.1)	20.60	<0.001
No blood drawn	115(55.3)	135(75.8)	86(48.6)	30.04	<0.001
No pain	78(37.5)	135(75.8)	51(28.8)	90.50	<0.001
Shorter time in awaiting results	75(36.1)	26(14.6)	70(39.5)	31.15	<0.001
All friends use	7(3.4)	5(2.8)	3(1.7)	1.05	0.592
Friends recommend	17(8.2)	9(5.1)	11(6.2)	1.57	0.456
Consultant recommend	28(13.5)	42(23.6)	21(11.9)	10.79	0.005
Concerns of oral fluid HIV rapid testing					
Had not ever heard	98(27.1)	208(51.4)	88(24.4)	75.61	<0.001
Testing accuracy	182(49.1)	171(42.2)	199(55.1)	12.78	0.002
Not free	28(7.5)	42(9.4)	23(6.4)	2.49	0.287
Acceptable price of oral fluid HIV rapid testing (USD)					
Median(Interquartile range)	6.5(3.0-11.3)	4.8(1.6-8.1)	8.1(2.4-16.1)	1.63 ^*^	0.018
Preferred test sites of oral fluid HIV rapid test					
General hospital	119(32.5)	85(21.0)	108(30.0)	14.33	0.001
Specialized hospital	58(15.8)	151(37.3)	60(16.7)	63.50	<0.001
Community hospital	51(13.9)	95(23.9)	64(17.8)	11.75	0.003
Community organization	30(8.2)	138(34.1)	36(10.0)	110.16	<0.001
CDC	236(64.5)	96(23.7)	162(5.0)	130.33	<0.001
Blood station	40(10.9)	27(6.7)	39(10.8)	5.44	0.066
Testing center	107(29.2)	74(18.3)	58(16.1)	21.85	<0.001
Self-test	105(28.1)	63(15.6)	117(32.5)	31.98	<0.001

* one-way analysis of variance

### Factors associated with willingness to accept oral fluid HIV rapid testing

Univariate analysis results of all variables among MSM, FSW and VCT clients were separately showed in the table S1, S2 and S3 of the support information. Factors with *P*-values below 0.10 that were considered for inclusion in the multivariable model for MSM were monthly income, education level, having ever heard of oral fluid HIV rapid testing, having ever taken oral fluid HIV rapid test, having ever taken an HIV test, having ever considered HIV home testing, considering HIV home testing using oral fluid HIV test kits (Table 4). For FSW, monthly income, having ever considered HIV home testing, considering HIV home testing using oral fluid HIV test kits, and engagement in HIV-related risk behaviors were included in the multivariable model (Table 5). For VCT clients, having ever heard of oral fluid HIV rapid testing, having ever taken an HIV test, considering HIV home testing using oral fluid HIV test kits were in all included with a *P*-value less than 0.10 (Table 6). 

**Table 4:** Factors associated with willingness to accept oral fluid HIV rapid testing among MSM in Shandong, China.

Factors ^*^	Willingness to accept the oral fluid HIV rapid testing		OR	AOR	95%CI	P-value
	Event/total	%				
Monthly income ($)						
≤645	237/334	71.0	1.0			
>645	33/37	89.2	3.38			
Having ever heard of oral fluid HIV rapid testing						
No	128/202	63.4	1.0			
Yes	142/169	84.0	3.04			
Having ever considered HIV home testing						
No	124/194	63.9	1.0			
Yes	146/177	82.5	2.66			
Educational level						
High school and lower	110/162	67.9	1.0	1.0		
College and higher	160/209	76.6	1.54	1.74	1.03-2.95	0.039
Having ever taken an HIV test						
No	70/122	57.4	1.0	1.0		
Yes	200/249	80.3	3.03	2.07	1.21-3.54	0.008
Having ever taken an oral fluid HIV rapid test						
No	223/323	69.0	1.0	1.0		
Yes	47/48	97.9	21.08	15.25	1.93-120.69	0.010
Considering HIV home testing using oral fluid HIV test kits						
No	84/155	54.2	1.0	1.0		
Yes	186/216	86.1	5.24	4.46	2.62-7.60	<0.001

* All factors in the table were variables with P<0.10 in univariate logistic regression models. Variables with AOR in the table were variables that eventually entered the multivariable logistic regression model for MSM group. OR, odds ratio; AOR, adjusted odds ratio; 95%CI, 95% confidence interval

**Table 5:** Factors associated with willingness to accept oral fluid HIV rapid testing among FSW in Shandong, China.

Factors ^*^	Willingness to accept the oral fluid HIV rapid testing		OR	AOR	95%CI	P-value
	Event/total	%				
Monthly income($)						
≤645	138/204	67.6	1.0			
>645	154/201	76.6	1.57			
Having ever considered HIV home testing						
No	225/325	69.2	1.0			
Yes	67/80	83.8	2.29			
HIV risk behaviors						
No	89/139	64.0	1.0	1.0		
Yes	203/266	76.3	1.81	1.68	1.01-2.77	0.044
Considering HIV home test using oral fluid HIV test kits						
No	117/198	59.1	1.0	1.0		
Yes	175/207	84.5	3.79	3.19	1.89-5.41	<0.001

* All factors in the table were variables with P<0.10 in univariate logistic regression models. Variables with AOR in the table were variables that eventually entered the multivariable logistic regression model for MSM group. OR, odds ratio; AOR, adjusted odds ratio; 95%CI, 95% confidence interval

**Table 6:** Factors associated with willingness to accept oral fluid HIV rapid testing among VCT clients in Shandong, China.

Factors *	Willingness to accept the oral fluid HIV rapid testing		OR	AOR	95%CI	P-value
	Event/total	%				
Having ever heard of oral fluid HIV rapid testing						
No	154/240	64.2	1.0			
Yes	90/121	74.4	1.62			
Having ever taken an HIV test						
No	120/208	57.7	1.0	1.0		
Yes	124/153	810	3.14	2.85	1.66-4.89	<0.001
Considering HIV home test using oral fluid HIV test kits						
No	80/167	47.9	1.0	1.0		
Yes	164/194	84.5	5.95	5.74	3.39-9.72	<0.001

* All factors in the table were variables with P<0.10 in univariate logistic regression models. Variables with AOR in the table were variables that eventually entered the multivariable logistic regression model for MSM group. OR, odds ratio; AOR, adjusted odds ratio; 95%CI, 95% confidence interval

Logistic regression showed that considering HIV home testing using oral fluid HIV test kits was independently associated with willingness to accept the oral fluid HIV test in all three groups, with AOR of 4.46, 3.19 and 5.74 respectively (Table 4,5,6). Besides this, independent predictors of willingness to accept the oral fluid HIV rapid testing for MSM included having ever taken oral fluid HIV rapid test (AOR= 15.25), having ever taken an HIV test (AOR= 2.07), and education level (AOR= 1.74) (Table 4). Factors associated with willingness to accept the test for FSW were engagement in HIV-related risk behaviors (AOR= 1.68) (Table 5). Having taken an HIV test (AOR= 2.85) was independently related to willingness to accept the testing among VCT clients (Table 6).

## Discussion

The results from our study showed that knowledge and experience of taking oral fluid HIV rapid test were limited among most-at-risk populations in Shandong Province. A large proportion of them had never heard of the testing and only 3.4% had used the oral fluid HIV rapid test during their most recent HIV test. However, most high-risk participants were willing to accept the oral fluid HIV rapid testing and there was no statistical difference between MSM, FSW and VCT clients. Previous studies conducted among most-at-risk populations and general populations showed that 64.5%-89.0% of participants preferred the oral fluid rapid HIV testing to traditional HIV testing [1,5,17,27,28,29]. Acceptance among MSM in our study (72.8%) was lower than the 85.1% acceptance rate in a recent study conducted in Beijing, China [1]. A possible reason may be unfamiliarity with the oral fluid rapid HIV testing, only 45.6% of MSM had ever heard of it in our study. The acceptance rate for FSW (72.1%) was similar to another study in rural India that reported 70% of women preferred to use oral fluid HIV rapid test over other HIV tests [17]. Similar results among VCT clients in our study were observed in a study conducted at a STD/HIV clinic with a 64.5% acceptance rate [27]. Another reason for the relatively low acceptance among most-at-risk populations in our study may be the low prevalence of HIV/AIDS in Shandong province. 

Not having to do a blood draw, no pain, and quick test results were three frequent reasons the most-at-risk populations were willing to accept oral fluid HIV rapid testing. Similar results were observed in previous studies where authors attributed preference for the oral fluid HIV rapid testing to its noninvasiveness, convenience, speed and ease of sample collection [17,22,26,30,31,32]. For FSW, 75.8% reported not having to do a blood draw and the pain-free aspect of the test to be primary reasons they were willingness to accept the oral fluid rapid HIV testing. This is consistent with a study conducted among women that found with venous blood draw, 20% of participants reported pain, 62% feared giving blood and 18% reported pain and swelling [17]. Some people may be more likely to accept the testing so as to avoid venipuncture. Besides that, shorter time in awaiting test results was also a very important reason for willing to test for HIV, especially among MSM and VCT clients [33]. Furthermore, a recent study showed that more than 90% participants preferred to receive their test results within one hour [29]. Hence, oral fluid rapid HIV test offers an alternative for individuals who desire no blood drawn, no pain and the ability to obtain results immediately to determine their sero-status. 

Our study indicated that considering to do HIV home testing using oral fluid HIV test kits was the common effect factor for willingness to accept oral fluid rapid HIV testing among MSM, FSW and VCT clients. Concerns about confidentiality, high visibility and emotional vulnerability of receiving results in public carry a barrier to HIV testing [16]. Oral fluid rapid HIV testing, similar to pregnancy testing, enables individuals to take an HIV test in the privacy of their home. Pant Pai et al pointed out that oral fluid rapid HIV testing was the only method of HIV testing that came close to being considered for home self-testing [9]. Furthermore, a study conducted in Singapore about the user-acceptability of self-HIV testing showed that the majority of respondents (89%) liked anonymous testing in private, 98% participants perceived the oral fluid-based HIV rapid test kit and found it convenient to use, and 95.8% reported its introductions were easy to understand [29]. Given these data, the oral fluid rapid HIV testing may be the perfect HIV testing method for most-at-risk populations opting to take home self-test. However, false positive results should be considered when using the home-based testing and when testing results are positive, appropriate HIV information and counseling should be provided in a timely manner. 

Having ever taken an HIV test was an independent predictor of willingness to accept oral fluid rapid HIV testing for MSM and VCT clients. Similar results were also observed in a recent study conducted in China wherein the author theorized that persons who had ever taken an HIV test may be knowledgeable about theoral fluid HIV rapid testing [1]. We also found that educational level was an independent predictor of willingness to accept the oral fluid HIV rapid testing among MSM, which was similar to a study conducted in Beijing, China [1]. Another study showed that lower education was a barrier to HIV testing [34]. Hence, more attention should be paid to MSM with lower educational attainment. However, education alone is not a solution. Having risk behaviors in the last three months was independently associated with willingness to accept an oral fluid rapid HIV test for FSW. These findings are similar with a previous study conducted in Southwest China indicating that acceptance of HIV-testing was strongly associated with high engagement in HIV-related risk behaviors, including inconsistent condom use with clients/stable partners, inconsistent condom use in the last three sex acts with clients/stable partners, intention of inconsistent condom use with clients, and drug abuse [35]. FSW who have high rates of engagement in HIV-related risk behaviors should be targeted for HIV prevention education in addition to offering oral fluid HIV testing.

Our study also explored the price point at which high-risk populations would be willing to purchase an oral fluid rapid HIV test. Lee et al showed that the price of the HIV test had the highest impact on willingness to accept an HIV testing, followed by the awaiting time to receive test results and test location [33]. Oraquick, the most popular CLIA-waived test, has also been criticized for its high costs (approximate cost US$ 17) [9]. A pilot study, sampling 240 HIV-positive patients, showed that most would pay no more than $15 USD for a home HIV test [36]. Our results showed that median price that participants would pay for the oral fluid HIV rapid testing was $6.5 USD, $4.8 USD and $8.1 USD among MSM, FSW and VCT clients respectively. These findings are similar to other studies in which participants were willing to pay no more than $8 USD for oral fluid HIV rapid testing [1,21,37]. The vast majority of respondents believed that the cost of oral fluid HIV rapid testing should be federally subsidized [37]. HIV testing using traditional methods is free in VCT clinics, but there is no government financial support for subsidizing oral fluid HIV rapid testing in China, and consumers must pay for the oral fluid tests out-of-pocket [1]. Therefore, high priced oral fluid HIV rapid testing may affect its uptake and scale-up in China. 

When assessing testing sites and venues, CDC was the most popular option for participants. The next best option preferred by MSM and VCT clients was general hospital. This finding differs from results in the U.S. where a physician’s office was the most common HIV testing site [38]. For FSW, a specialized hospital was their favorite testing site. A study in Taiwan suggested that pharmacies would be the most appropriate and accessible venue for selling the oral fluid HIV rapid testing kits [37]. Based on those data and our study, findings suggest that the test kits should be available in a variety of health facilities in order to expand the oral fluid HIV rapid testing.

Despite the concerns about credibility among participants in this study, and the existing controversy about the accuracy of oral fluid HIV rapid testing [5,39,40], there is still an epidemic imperative to support the expansion of oral fluid HIV rapid testing in China. The use of noninvasive and rapid HIV testing with rapid response time is critical to the early identification of HIV status [41]. The anti-HIV test strip methodology for saliva specimens is rapid, reliable and easy to perform and interpret [42]. Saliva specimens can be readily collected from any individual, and there is a reduction in hazard risk [42]. Thus, the oral fluid HIV rapid testing is a potential alternative for individuals to do home self-testing, avoids the issue of stigma, and prevents visibility in public settings. 

Our study has some limitations. First, the three most-at-risk populations were not easy to sample, hence our data was limited in that participants at our study site may not be representatives of similar risk groups in other cities. Second, participants were asked to state a preference for a particular type of testing before trying an oral fluid HIV rapid test. Therefore, results regarding which testing type participants preferred may have been biased by the order in which we discussed options for HIV testing and then used the oral fluid HIV rapid test. Third, this investigation addressed sensitive questions that may have led to misreporting of personal attitudes and behaviors due to social desirability bias. Thus, further research is needed to explore the acceptance of the oral fluid HIV rapid testing among users.

In conclusion, although knowledge and experience were limited, the oral fluid HIV rapid testing had a high acceptability rate among most-at-risk populations in China. Oral fluid HIV rapid testing provides a potential alternative for individuals who do not test due to privacy concerns in that home self-testing may be a viable option to increase the rate of HIV screening among most-at-risk populations. Appropriate pricing, safe and anonymous testing venues, and increased knowledge to address concerns about the accuracy and safety of the oral fluid HIV rapid testing may be effective strategies for increasing acceptability among most-at-risk populations. 

## Supporting Information

Table S1 **Associations between willingness to accept oral fluid HIV rapid test and socio-demographic characteristics, sexual behaviors and HIV testing history among MSM in Qingdao and Yantai cities, Shandong province, China.**
(DOCX)

Table S2 **Associations between willingness to accept oral fluid HIV rapid test and socio-demographic characteristics, sexual behaviors, HIV testing history among FSW in Qingdao and Zibo cities, Shandong province, China.**
(DOCX)

Table S3 **Associations between willingness to accept oral fluid HIV rapid test and socio-demographic characteristics, sexual behaviors, HIV testing history among VCT clients in Qingdao and Yantai cities, Shandong province, China.**
(DOCX)
