# Supplementary material for: Factors Associated with Willingness to Accept Oral Fluid HIV Rapid Testing among Most-at-Risk Populations in China
Source: PLoS One. 2013 Nov 19;8(11):e80594. doi: 10.1371/journal.pone.0080594 (PMC3834295; doi:10.1371/journal.pone.0080594)
Supplement: Table S1 — Associations between willingness to accept oral fluid HIV rapid test and socio-demographic characteristics, sexual behaviors and HIV testing history among MSM in Qingdao and Yantai cities, Shandong province, China. (DOCX) [file pone.0080594.s001.docx]

**Table S1** Associations between willingness to accept oral fluid HIV rapid test and socio-demographic characteristics, sexual behaviors and HIV testing history among MSM in Qingdao and Yantai cities, Shandong province, China

| Variables | Willing to accept oral fluid HIV rapid test | | | OR | 95%CI | P-value |
| --- | --- | --- | --- | --- | --- | --- |
|  | Event/total | | % |  |  |  |
| Age (years) |  | |  |  |  |  |
| ≤25 | 144/198 | | 72.7 | 1.0 |  |  |
| >25 | 126/173 | | 72.8 | 1.01 | 0.64-1.59 | 0.98 |
| Education level |  | |  |  |  |  |
| High school or lower | 110/162 | | 67.9 | 1.0 |  |  |
| College or higher | 160/209 | | 76.6 | 1.54 | 0.98-2.44 | 0.06 |
| Monthly income($) |  | |  |  |  |  |
| ≤645 | 237/334 | | 71.0 | 1.0 |  |  |
| >645 | 33/37 | | 89.2 | 3.38 | 1.17-9.79 | 0.03 |
| Occupation |  | |  |  |  |  |
| Others | 18/27 | | 66.7 | 1.0 |  | 0.32 |
| Business service | 64/81 | | 79.0 | 1.88 | 0.72-4.93 | 0.20 |
| Student | 55/77 | | 71.4 | 1.25 | 0.49-3.20 | 0.64 |
| Workers | 56/75 | | 74.7 | 1.47 | 0.57-3.83 | 0.43 |
| Food and beverage workers | 44/61 | | 72.1 | 1.29 | 0.49-3.44 | 0.61 |
| Cadres staff | 10/19 | | 52.6 | 0.56 | 0.17-1.85 | 0.34 |
| Teacher | 4/8 | | 50.0 | 1.50 | 0.25-8.98 | 0.66 |
| nanny/housewife/unemployment | 13/15 | | 86.7 | 0.50 | 0.10-2.48 | 0.40 |
| Farmer/fisher/migrant workers | 18/27 | | 66.7 | 3.25 | 0.60-17.62 | 0.17 |
| Having ever taken an HIV test |  | |  |  |  |  |
| No | 70/122 | | 57.4 | 1.0 |  |  |
| Yes | 200/249 | | 80.3 | 3.03 | 1.88-4.88 | 0.003 |
| Having ever heard of oral fluid HIV rapid test | | | | | | |
| No | 128/202 | | 63.4 | 1.0 |  |  |
| Yes | 142/169 | | 84.0 | 3.04 | 1.84-5.02 | <0.001 |
| Having ever taken oral fluid HIV rapid test | | | | | | |
| No | 223/323 | | 69.0 | 1.0 |  |  |
| Yes | 47/48 | | 97.9 | 21.08 | 2.87-154.91 | 0.003 |
| Having ever considered HIV home testing | | | | | | |
| No | 124/194 | | 63.9 | 1.0 |  |  |
| Yes | 146/177 | | 82.5 | 2.66 | 1.64-4.32 | <0.001 |
| Considered HIV home testing using oral fluid HIV test kits | | | | | | |
| No | 84/155 | | 54.2 | 1.0 |  |  |
| Yes | 186/216 | | 86.1 | 5.24 | 3.18-8.63 | <0.001 |
| HIV risk behaviors |  |  | |  |  |  |
| No | 33/41 | 80.5 | | 1.0 |  |  |
| Yes | 237/330 | 71.8 | | 0.62 | 0.28-1.39 | 0.24 |
