# Supplementary material for: Factors Associated with Willingness to Accept Oral Fluid HIV Rapid Testing among Most-at-Risk Populations in China
Source: PLoS One. 2013 Nov 19;8(11):e80594. doi: 10.1371/journal.pone.0080594 (PMC3834295; doi:10.1371/journal.pone.0080594)
Supplement: Table S2 — Associations between willingness to accept oral fluid HIV rapid test and socio-demographic characteristics, sexual behaviors, HIV testing history among FSW in Qingdao and Zibo cities, Shandong province, China. (DOCX) [file pone.0080594.s002.docx]

**Table S2** Associations between willingness to accept oral fluid HIV rapid test and socio-demographic characteristics, sexual behaviors, HIV testing history among FSW in Qingdao and Zibo cities, Shandong province, China

| Variables | Willing to accept oral fluid HIV rapid test | | | OR | 95%CI | P-value |
| --- | --- | --- | --- | --- | --- | --- |
|  | Event/total | | % |  |  |  |
| Age (years) |  | |  |  |  |  |
| ≤25 | 191/264 | | 72.3 | 1.0 |  |  |
| >25 | 101/141 | | 71.6 | 0.97 | 0.61-1.52 | 0.88 |
| Education level |  | |  |  |  |  |
| High school or lower | 276/385 | | 71.7 | 1.0 |  |  |
| College or higher | 16/20 | | 80.0 | 1.58 | 0.52-4.83 | 0.42 |
| Monthly income($) |  | |  |  |  |  |
| ≤645 | 138/204 | | 67.6 | 1.0 |  |  |
| >645 | 154/201 | | 76.6 | 1.57 | 1.01-2.43 | 0.045 |
| Occupation* |  | |  |  |  |  |
| Others | 37/52 | | 71.2 | 1.0 |  |  |
| Business service | 255/353 | | 72.2 | 1.06 | 0.55-2.01 | 0.87 |
| Having ever taken an HIV test |  | |  |  |  |  |
| No | 116/167 | | 69.5 | 1.0 |  |  |
| Yes | 176/238 | | 73.9 | 1.25 | 0.81-1.94 | 0.32 |
| Having ever heard of oral fluid HIV rapid test | | | | | | |
| No | 261/366 | | 71.3 | 1.0 |  |  |
| Yes | 31/39 | | 79.5 | 1.56 | 0.69-3.50 | 0.28 |
| Having ever taken oral fluid HIV rapid test | | | | | | |
| No | 280/392 | | 71.4 | 1.0 |  |  |
| Yes | 12/13 | | 92.3 | 4.8 | 0.62-37.35 | 0.13 |
| Having ever considered HIV home testing | | | | | | |
| No | 225/325 | | 69.2 | 1.0 |  |  |
| Yes | 67/80 | | 83.8 | 2.29 | 1.21-4.34 | 0.011 |
| Considered HIV home testing using oral fluid HIV test kits | | | | | | |
| No | 117/198 | | 59.1 | 1.0 |  |  |
| Yes | 175/207 | | 84.5 | 3.79 | 2.36-6.07 | <0.001 |
| HIV risk behaviors |  |  | |  |  |  |
| No | 89/139 | | 64.0 | 1.0 |  |  |
| Yes | 203/266 | | 76.3 | 1.81 | 1.16-2.83 | 0.009 |

*Because of the data of students, workers, food and beverage workers, cadres staff, teacher, nanny, farmers and unemployment are few, so I combined these as the others.
