# Supplementary material for: Factors Associated with Willingness to Accept Oral Fluid HIV Rapid Testing among Most-at-Risk Populations in China
Source: PLoS One. 2013 Nov 19;8(11):e80594. doi: 10.1371/journal.pone.0080594 (PMC3834295; doi:10.1371/journal.pone.0080594)
Supplement: Table S3 — Associations between willingness to accept oral fluid HIV rapid test and socio-demographic characteristics, sexual behaviors, HIV testing history among VCT clients in Qingdao and Yantai cities, Shandong province, China. (DOCX) [file pone.0080594.s003.docx]

**Table S3** Associations between willingness to accept oral fluid HIV rapid test and socio-demographic characteristics, sexual behaviors, HIV testing history among VCT clients in Qingdao and Yantai cities, Shandong province, China

| Variables | Willing to accept oral fluid HIV rapid test | | | OR | 95%CI | P-value |
| --- | --- | --- | --- | --- | --- | --- |
|  | Event/total | | % |  |  |  |
| Age (years) |  | |  |  |  |  |
| ≤25 | 85/130 | | 65.4 | 1.0 |  |  |
| >25 | 159/231 | | 68.8 | 1.17 | 0.74-1.85 | 0.50 |
| Education level |  | |  |  |  |  |
| High school or lower | 96/138 | | 69.6 | 1.0 |  |  |
| College or higher | 148/223 | | 66.4 | 0.86 | 0.55-1.36 | 0.53 |
| Monthly income($) |  | |  |  |  |  |
| ≤645 | 218/322 | | 67.7 | 1.0 |  |  |
| >645 | 26/39 | | 66.7 | 0.95 | 0.47-1.93 | 0.90 |
| Occupation |  | |  |  |  |  |
| Others | 14/24 | | 58.3 | 1.0 |  | 0.022 |
| Business service | 49/64 | | 76.6 | 2.33 | 0.86-6.32 | 0.096 |
| Student | 42/54 | | 77.8 | 1.27 | 0.49-3.32 | 0.62 |
| Workers | 51/95 | | 53.7 | 2.50 | 0.89-7.03 | 0.083 |
| Food and beverage workers | 21/29 | | 72.4 | 0.83 | 0.34 | 2.05 |
| Cadres staff | 10/12 | | 83.3 | 1.88 | 0.59-5.92 | 0.28 |
| Teacher | 8/10 | | 80.0 | 3.57 | 0.64-19.97 | 0.15 |
| nanny/housewife/unemployment | 8/9 | | 88.9 | 2.86 | 0.50-16.43 | 0.24 |
| Farmer/fisher/migrant workers | 14/24 | | 58.3 | 5.71 | 0.61-53.23 | 0.13 |
| Having ever taken an HIV test |  | |  |  |  |  |
| No | 120/208 | | 57.7 | 1.0 |  |  |
| Yes | 124/153 | | 81.0 | 3.14 | 1.92-5.11 | <0.001 |
| Having ever heard of oral fluid HIV rapid test | | | | | | |
| No | 154/240 | | 64.2 | 1.0 |  |  |
| Yes | 90/121 | | 74.4 | 1.62 | 1.0-2.64 | 0.05 |
| Having ever taken oral fluid HIV rapid test | | | | | | |
| No | 10/18 | | 55.6 | 1.0 |  |  |
| Yes | 234/343 | | 68.2 | 1.01 | 0.22-1.52 | 0.27 |
| Having ever considered HIV home testing | | | | | | |
| No | 159/245 | | 64.9 | 1.0 |  |  |
| Yes | 85/116 | | 73.3 | 1.48 | 0.91-2.42 | 0.11 |
| Considered HIV home testing using oral fluid HIV test kits | | | | | | |
| No | 80/167 | | 47.9 | 1.0 |  |  |
| Yes | 164/194 | | 84.5 | 5.95 | 3.63-9.74 | <0.001 |
| HIV risk behaviors |  |  | |  |  |  |
| No | 35/48 | | 72.9 | 1.0 |  |  |
| Yes | 209/313 | | 66.8 | 0.75 | 0.38-1.47 | 0.40 |
